# Integrated bioinformatics analysis identified leucine rich repeat containing 15 and secreted phosphoprotein 1 as hub genes for calcific aortic valve disease and osteoarthritis

**DOI:** 10.1049/syb2.12091

**Published:** 2024-04-02

**Authors:** Shuji Gong, Kun Xiang, Le Chen, Huanwei Zhuang, Yaning Song, Jinlan Chen

**Affiliations:** ^1^ Department of Cardiovascular Surgery The Second Xiangya Hospital of Central South University Changsha Hunan China

**Keywords:** bioinformatics, cardiology, data analysis

## Abstract

Calcific aortic valve disease (CAVD) and osteoarthritis (OA) are common diseases in the ageing population and share similar pathogenesis, especially in inflammation. This study aims to discover potential diagnostic and therapeutic targets in patients with CAVD and OA. Three CAVD datasets and one OA dataset were obtained from the Gene Expression Omnibus database. We used bioinformatics methods to search for key genes and immune infiltration, and established a ceRNA network. Immunohistochemical staining was performed to verify the expression of candidate genes in human and mice aortic valve tissues. Two key genes obtained, leucine rich repeat containing 15 (LRRC15) and secreted phosphoprotein 1 (SPP1), were further screened using machine learning and verified in human and mice aortic valve tissues. Compared to normal tissues, the infiltration of immune cells in CAVD tissues was significantly higher, and the expressions of LRRC15 and SPP1 were positively correlated with immune cells infiltration. Moreover, the ceRNA network showed extensive regulatory interactions based on LRRC15 and SPP1. The authors’ findings identified LRRC15 and SPP1 as hub genes in immunological mechanisms during CAVD and OA initiation and progression, as well as potential targets for drug development.

## Graphical abstract

The authors’ study identified leucine rich repeat containing 15 (LRRC15) and secreted phosphoprotein 1 (SPP1) as hub genes in calcific aortic valve disease (CAVD) and osteoarthritis (OA) and found that they were related to immune processes. This offers new perspectives on the pathogenesis and treatment of CAVD and OA treatment.

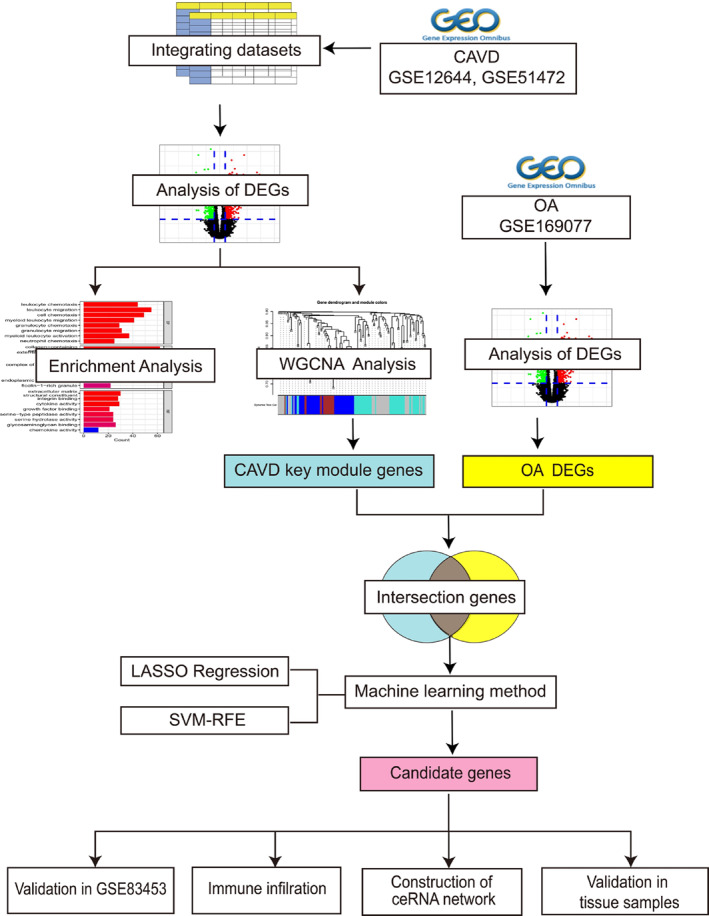

## 1 INTRODUCTION

Calcific aortic valve disease (CAVD) and osteoarthritis (OA) are common diseases and significant causes of morbidity and mortality among ageing populations worldwide [1, 2, 3, 4, 5, 6, 7]. However, no effective medications are available to limit or prevent CAVD and OA development, although surgical repair is an optimal therapeutic strategy with considerable residual morbidity and mortality risks [8, 9]. Thus, an urgent clinical need exists to further undercover the underlying mechanisms and development of potential novel therapeutic medications.

Accumulating evidence demonstrated that inflammation contributed importantly to the pathogenesis of CAVD and OA [9, 10, 11]. For example, an inflammatory response initiated by aortic valve endothelial cell dysfunction is the early pathological change of CAVD [12]. At this stage, injured valve endothelial cells released multiple chemokines to recruit various circulating leucocytes (such as monocytes, macrophages, neutrophils and T cells) and facilitated those inflammatory cells infiltrated into the aortic valve and activation [13]. Moreover, activated inflammatory cells were also found in the calcific aortic valve leaflet and play critical roles in mediating valve fibrosis and stenosis [14, 15, 16]. Like CAVD, inflammation exhibited a great impact on OA development and progression, and there are considerable immune cells, especially macrophages, accumulated in the synovium of OA patients [9, 17, 18]. Furthermore, targeting inflammatory processes hold great promising in improving aortic valve calcification or OA [19, 20]. Recently, emerging studies indicated that bioinformatics analysis concomitant or common diseases that share similar pathogenic mechanism could provide a new direction for developing a novel therapeutic strategy [21, 22]. Yet, little studies focus on CAVD and OA to discover potential therapeutic targets for CAVD and OA treatment, especially in inflammation.

**FIGURE 1:**
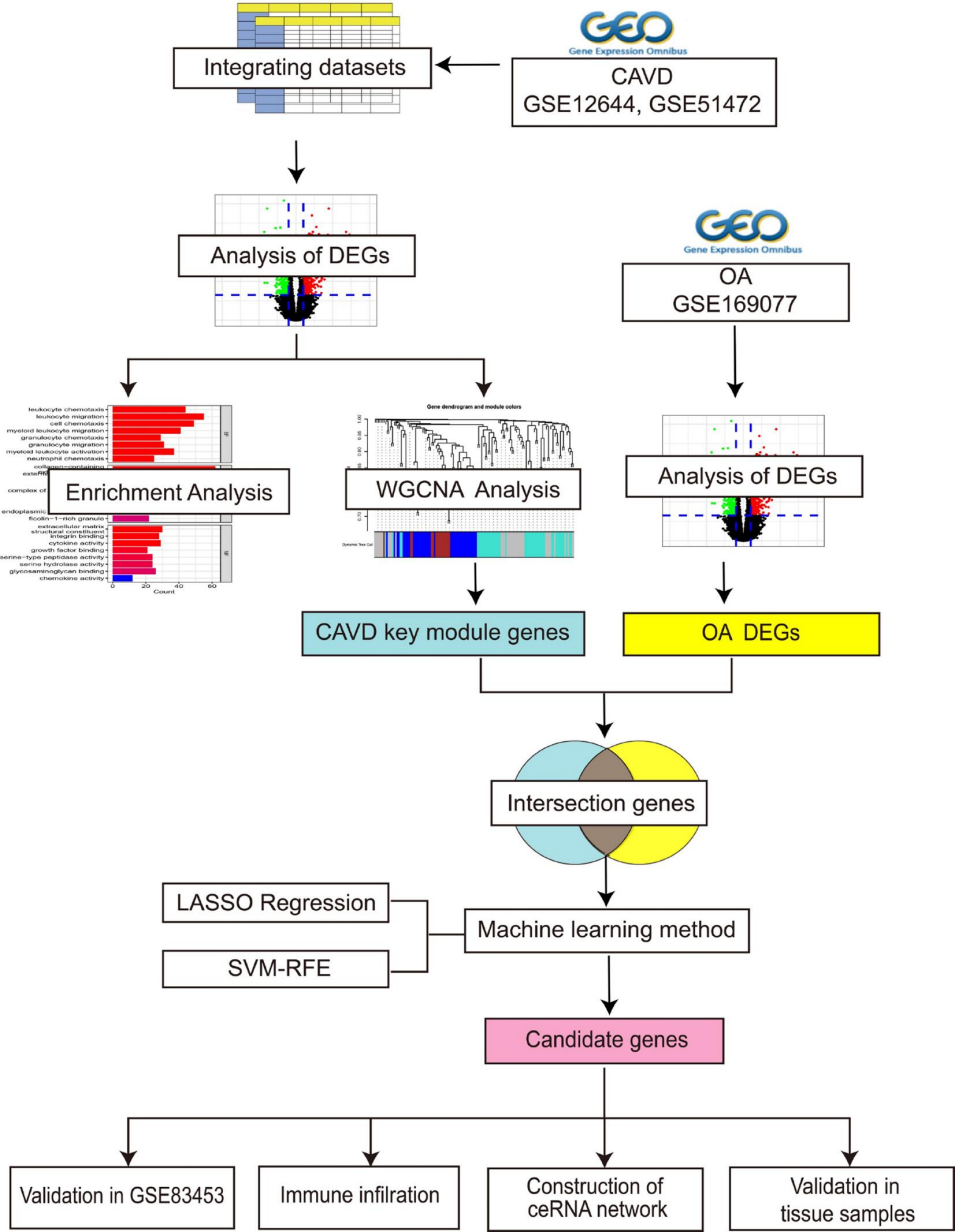
Research flow chart. DEGs, differentially expressed genes; WGCNA, weighted gene co‐expression network analysis; LASSO, least absolute shrinkage selection operator; SVM‐RFE, support vector machine‐recursive feature elimination.

In the current study, we analysed two CAVD datasets and one OA dataset downloaded from the Gene Expression Omnibus (GEO) database for common differentially expressed genes (DEGs) and combined used weighted gene co‐expression network analysis (WGCNA) and machine learning methods to identify immune‐related hub genes from those DEGs. Moreover, we verified those hub genes in another CAVD datasets and human and mice calcific aortic valve leaflets (Figure 1 shows the current study flow chart.). This work will provide new insights and direction for further understanding the mechanisms of CAVD and OA and will help for novel medications development.

## 2 MATERIALS AND METHODS

### 2.1 Microarray datasets information and processing

CAVD microarray datasets (GSE12644, GSE51472, and GSE83453) were obtained from the GEO database (http://www.ncbi.nlm.nih.gov/geo). Specifically, the GSE12644 dataset included 10 normal aortic valves and 10 calcified aortic valves, GSE51472 contains five normal samples and five calcified aortic valve samples, and GSE83453 included nine calcified tricuspid aortic valve samples and eight normal samples. The GSE169077 dataset was an OA gene expression profile and included five normal and six OA samples. We log2‐transformed the expression levels of GSE12644 and GSE51472, the batch correction was performed using the package SVA (3.50.0) and then normalised the merged data rows for subsequent analysis. The same method was used to analyse GSE169077. The GSE83453 dataset was used as a validation cohort to test the results obtained from the merge data.

### 2.2 Identification of differentially expressed genes

Limma package (3.58.1) was used to generate the DEGs from CAVD and OA GEO datasets. For CAVD, the merge date with adjusted *p* values < 0.05 and |log2FC| > 0.5. For OA, we obtained DEGs with *p*‐values <0.05, and |log2FC| > 0.5. The DEG data were processed to draw heat maps using the pheatmap package (1.0.12).

### 2.3 Pathway enrichment analysis of DEGs

To explore the biological meaning of DEGs in CAVD, all DEGs were subjected to gene ontology (GO) and Kyoto Encyclopaedia of Genes and Genomes (KEGG) pathway enrichment analysis using the clusterProfiler package (4.10.0), with the threshold of *p* value < 0.05 and *q* value < 0.05.

### 2.4 WGCNA

WGCNA is an algorithm that can be used to identify clusters (modules) of related genes. The WGCNA package (1.72–1) was used to establish a co‐expression network for the top 25% of DEGs [23, 24]. Using this algorithm, we obtained the best power value by testing different scales of independence and mean connectivity (power values ranging from 1 to 20). The power value was considered the best when the degree of independence was set to 0.85. The adjacency matrix was converted to obtain the topological overlap matrix (TOM) and corresponding dissimilarity (1‐TOM). Subsequently, the minimum number of module genes was set at 10 after similar genes were clustered. The most relevant module was used as the key module for the subsequent analysis. To analyse the relationship between CAVD and OA, we identified common genes for the key modules of CAVD and DEGs of OA.

### 2.5 Screening hub genes by machine learning

Hub genes were obtained from the common genes of CAVD and OA through two machine learning methods: least absolute shrinkage selection operator (LASSO) and support vector machine‐recursive feature elimination (SVM‐RFE), respectively. We used the intersection genes of CAVD key modules and OA DEGs to construct LASSO and SVM‐RFE algorithms through the glmnet package (4.1–8) and e1071 package (1.17–13). Hub genes were obtained by identifying common genes between the two machine learning methods. The expression levels of hub genes in the CAVD samples and healthy individuals were assessed using box plots. The diagnostic ability of hub genes was examined using the area under the ROC curve (AUC). Finally, hub genes were examined using GSE83453.

### 2.6 Analysis of immune cell infiltration

We derived the enrichment scores for each immune‐related cell type using the (single‐sample gene set enrichment analysis) ssGSEA method. The ssGSEA function in the GSVA package (1.50.0) was used to calculate the infiltration level of the immune cell [25]. The vioplot package (0.4.0) was used to compare immune cell levels between the disease and control groups [26]. The relationship between the expression of hub genes and the degree of immune cell infiltration was examined using Spearman's correlation.

### 2.7 ceRNA network construction

To predict the relationship of lncRNA‐miRNA‐mRNA, we constructed a ceRNA network. We used starBase database to predict the miRNAs interacting with the mRNAs of hub genes. Next, we downloaded the mRNA sequences of hub genes from the National Centre for Biotechnology Information (NCBI) and obtained the human miRNA sequences from miRbase. Miranda software is used to predict miRNA, and the miRNA‐mRNA binding score greater than 160 was considered effective. Furthermore, to acquire the ceRNA network, we searched the expected miRNA in starBase and filtered miRNA‐lncRNA. Finally, Cytoscape was used to visualise the obtained results.

### 2.8 CAVD animal models

Our animal experiments follow the Guide for the Care and Use of Laboratory Animals published by the US National Institutes of Health and were approved by the Institutional Animal Care and Use Committee at the Second Xiangya Hospital of Central South University (Protocol No. 2021810). Male *Apoe* ^*−/−*^ (*n* = 8) nd C57BL/6 mice (*n* = 4) from 8 to 10 weeks old were purchased from Beijing Vital River Laboratory Animal Technology Co. in China. All mice were housed under a 12 h light/dark cycle in a pathogen‐free animal facility with free access to food and water. Mice were kept on a standard chow diet or on a 1.25% high cholesterol diet (HCD; D12108C, Research Diets) for 24 weeks to produce CAVD. Then, mice were humanely sacrificed with carbon dioxide narcosis, and aortic roots were harvested and embedded in an optimum cutting temperature (OCT) compound or frozen at −80°C.

### 2.9 Patient sample collection

The use of discarded and de‐identified human aortic valve specimens (protocol No. 82100491) was approved by the Institution Research Ethics Committee of Second Xiangya Hospital of Central South University and conducted under the guidance of the Declaration of Helsinki. Informed consent was obtained from each patient before the collection of samples. Aortic valve samples were obtained from six CAVD patients and four healthy organ donors during surgery. After aortic valve samples were collected, formalin fixation and paraffin embedding were performed using standard methods.

#### 2.9.1 Immunohistochemical staining

Immunohistochemical staining was performed as described previously [27, 28]. Briefly, serial cryostat sections (6 *μ*m) from mice or paraffin sections (6 *μ*m) from human aortic valves were prepared and processed for immunostaining to detect leucine rich repeat containing 15 (LRRC15) (1:100, E‐AB‐15178, Elabscience) and secreted phosphoprotein 1 (SPP1) (1:100, 22952‐1‐AP, Proteintech). Immunohistochemical images were captured by Nikon Ti2 295 microscopy (Nikon). The LRRC15‐ and SPP1‐positive areas within the aortic valve were determined by detecting the staining intensity with computer‐assisted image analysis software (Image‐Pro Plus; Media Cybernetics), and the data were presented as positive area per *μ*m [2] of the aortic valve area.

### 2.10 Statistical analysis

GraphPad Prism software (Version 8.4, GraphPad Software, Inc), or R Studio, was used for statistical analysis. The unpaired 2‐tailed Student's *t* test was used to examine the statistical significance between two groups with normally distributed continuous variables. For data without normal distribution, the non‐parametric Mann–Whitney *U* test was used to compare two groups. All data are presented as mean ± SEM, and *p* < 0.05 was considered statistically significant.

## 3 RESULTS

### 3.1 Data processing and identification of DEGs

**FIGURE 2:**
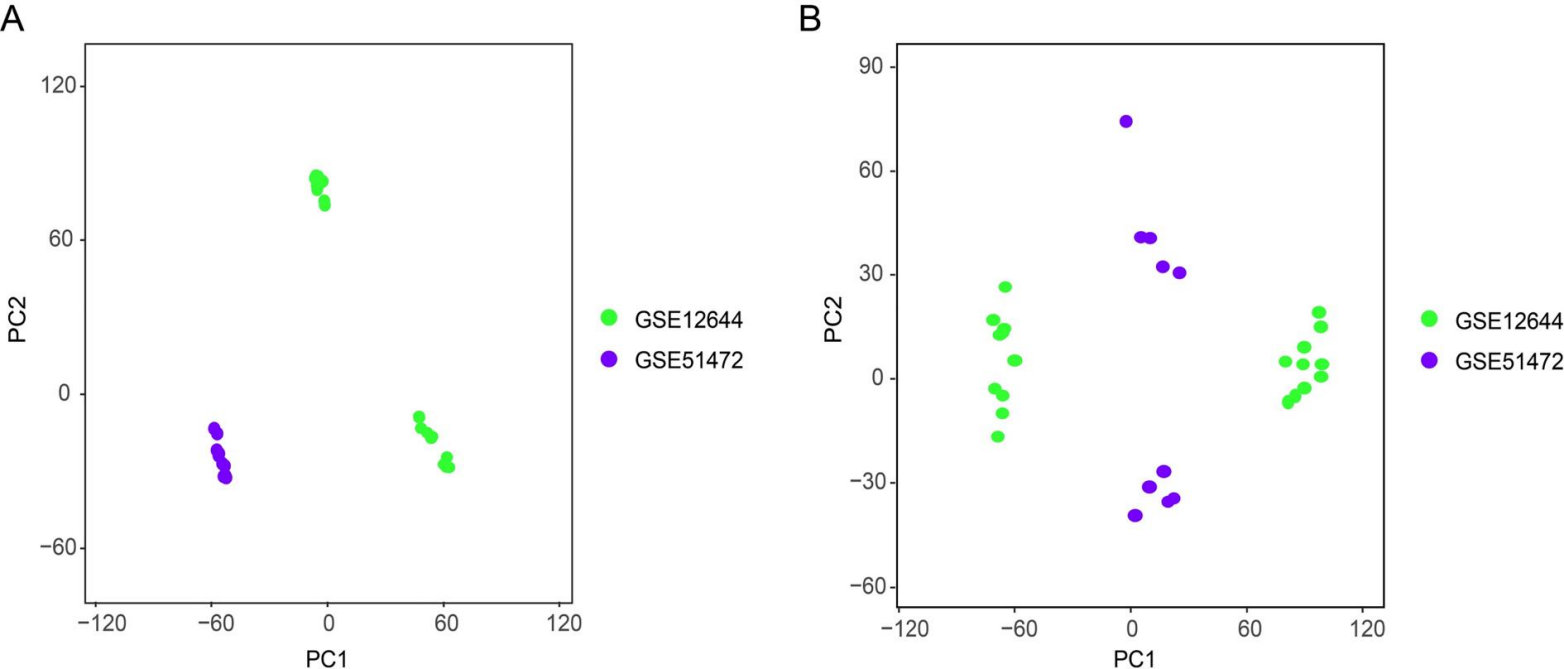
Cluster plot of PCA analysis of gene expression datasets. (a) PCA clustering results of GSE12644 and GSE51472 before sample correction and elimination of batch effects. (b) PCA clustering results of GSE12644 and GSE51472 after sample correction and elimination of batch effects. Each dot represents a sample, green dots represent GSE12644 samples and purple dots represent GSE51472 samples. PCA, principal component analysis.

**FIGURE 3:**
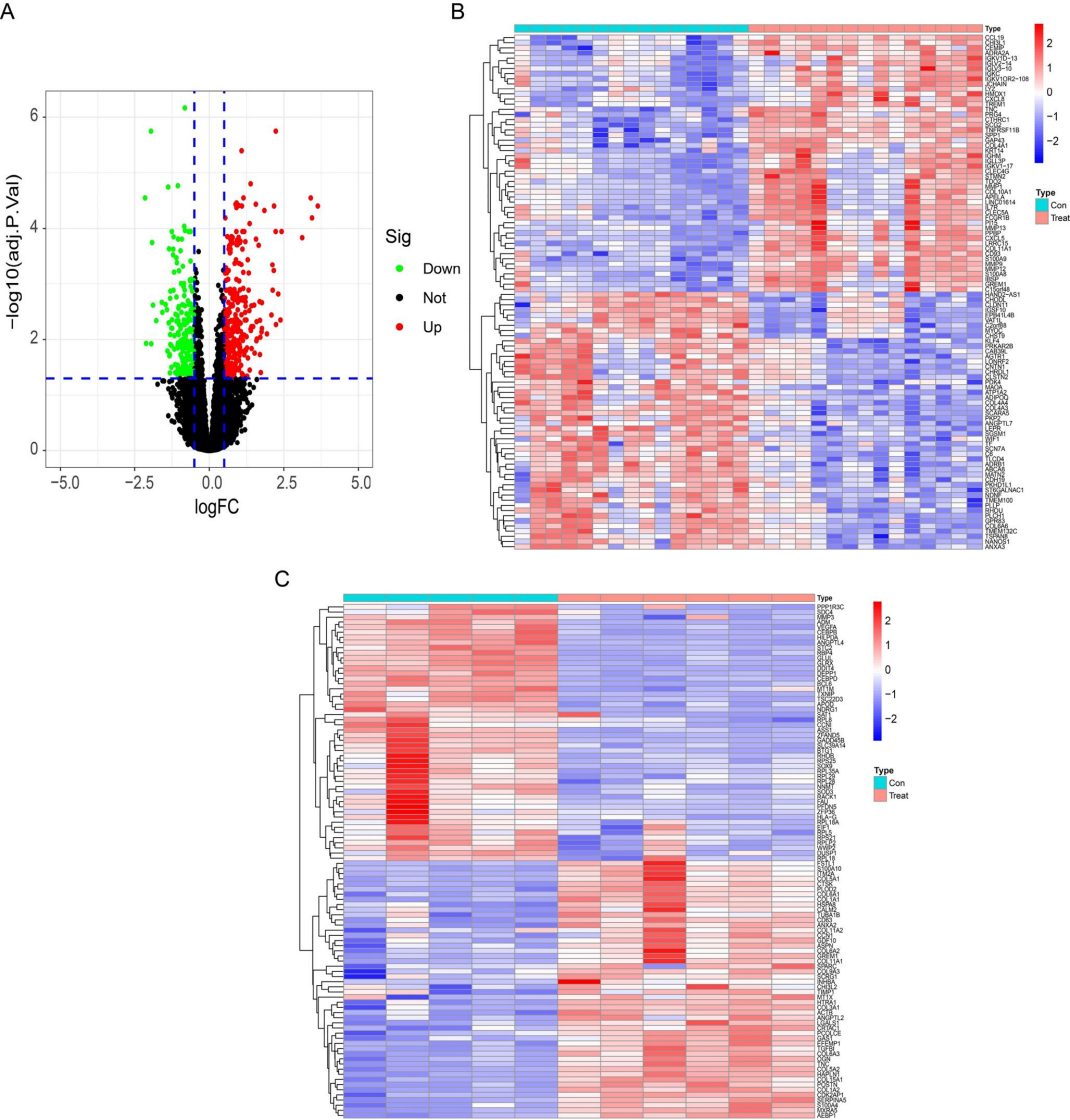
Differential genes for CAVD and OA. (a) Volcano plot of DEGs of CAVD; each point represents a gene, red represents up‐regulated differential genes, black represents no significant differential genes, and the green represents down‐regulated differential genes. (b) Cluster heatmap of CAVD DEGs expression between normal and disease groups. Cyan represents CAVD groups, red‐orange represents normal groups. (c) Cluster heatmap of OA DEGs expression between normal and disease groups. CAVD, calcific aortic valve disease; DEGs, differentially expressed gene; OA, osteoarthritis.

To adjust the batch effects of the merged CAVD GEO datasets (GSE12644 and GSE51472) (Figure 2a), batch correction using R package SVA was applied to those two datasets, and the PCA cluster diagram exhibited an efficient effect of removing batch effects (Figure 2b), indicating that the merged datasets can be further processed to avoid the analysis error. By using the Limma package, we obtained 554 DEGs (adjusted *p* values < 0.05 and log2FC > 0.5) in merged CAVD GEO datasets (Figure 3a,b). Moreover, among those 554 DEGs, 311 genes were upregulated and 233 genes were downregulated (Figure 3a). Furthermore, we obtained 1086 DEGs (*p* values < 0.05 and |log2FC| > 0.5) in OA datasets (GSE169077) (Figure 3c).

### 3.2 Enrichment analysis of DEGs in CAVD

**FIGURE 4:**
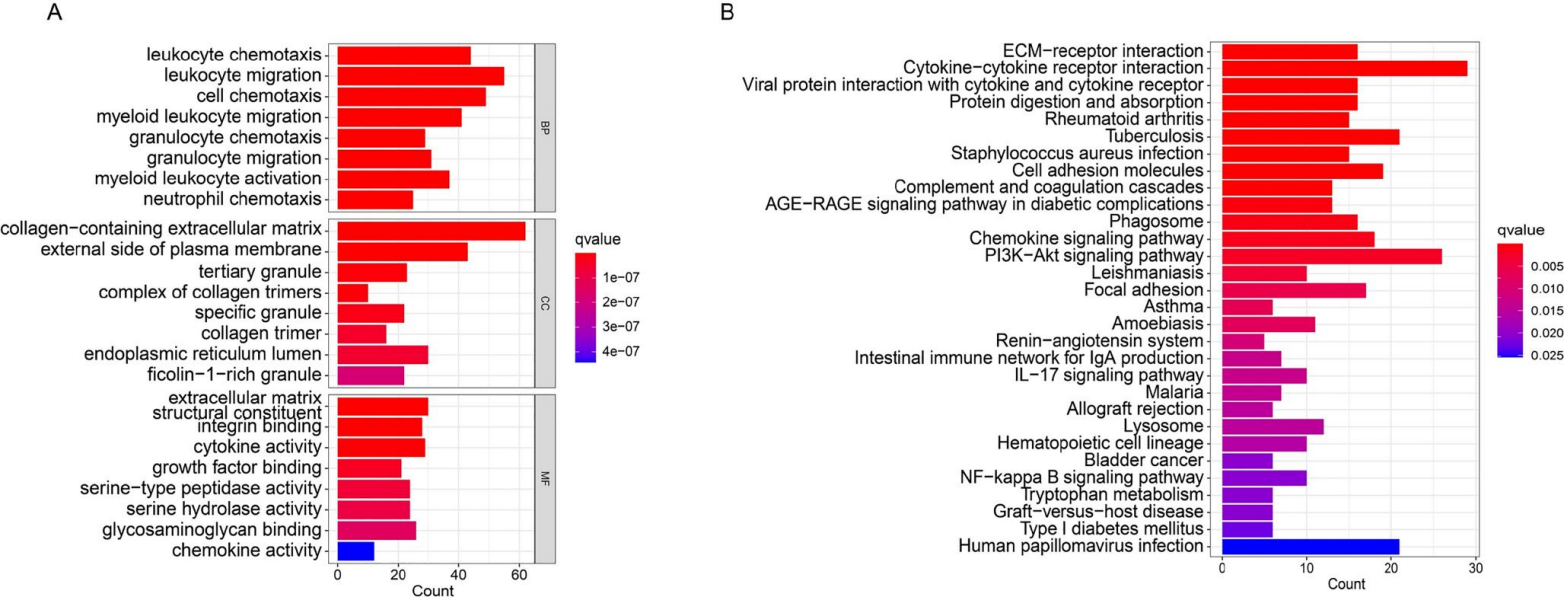
Functional enrichment analysis of DEGs in calcific aortic valve disease. (a) Gene ontology enrichment analysis results. The *x*‐axis represents the number of genes that are functionally enriched, and the *y*‐axis represents the enriched pathways. BP, biological process; CC, cellular component; MF, molecular function; (b) Kyoto encyclopedia of genes and genomes enrichment analysis of the top 30 pathways results. The *x*‐axis represents the number of genes that are functionally enriched, and the *y*‐axis represents the enriched pathways.

To understand the biological functions of those DEGs in merged CAVD GEO datasets (Figure 3), R package clusterProfiler was used to perform the GO and KEGG pathway enrichment analyses. As shown in Figure 4a, GO analysis showed that those DEGs were significantly related to inflammatory processes, including leucocyte chemotaxis, leucocyte migration, myeloid leucocyte migration, myeloid leucocyte activation and cell chemotaxis, for the biological process (BP). Collagen‐containing extracellular matrix, external side of plasma membrane, tertiary granule, complex of collagen trimers and specific granule were the most enriching cellular component (CC) among those DEGs (Figure 4a). For the molecular function (MF), DEGs were mainly related to extracellular matrix structural constituents, integrin binding, cytokine activity, and chemokine activity (Figure 4a). In line with GO enrichment analyses, KEGG pathway enrichment analyses also exhibited that DEGs were most enriched in inflammation‐associated pathways among the top 30 gene sets, including cytokine‐cytokine receptor interaction, viral protein interaction with cytokine and cytokine receptors, cell adhesion molecules, chemokine signalling pathway, IL‐17 signalling pathway, and NF‐kappa B signalling pathway (Figure 4b). Collectively, these results indicate that DEGs in merged CAVD GEO datasets were strongly associated with inflammatory responses.

### 3.3 The key co‐expression networks and modules in CAVD

**FIGURE 5:**
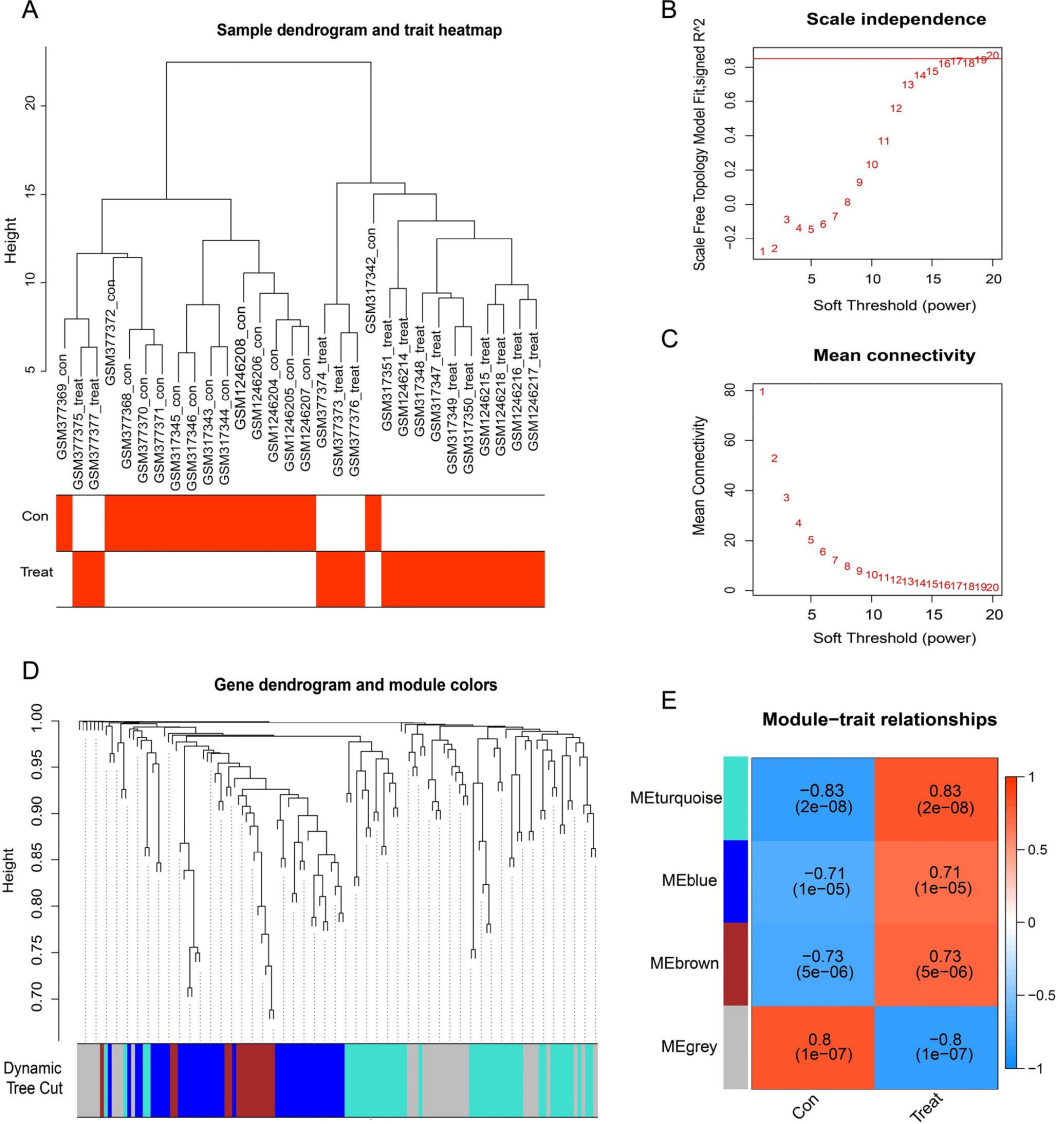
Weighted gene co‐expression network analysis results. (a) Sample clustering dendrogram for CAVD. (b) Scale‐free topology model fit corresponds to different powers, representing the scale independence of the analysis. (c) Mean connectivity corresponds to different powers. (d) Dendrogram of dissimilarity metric (1‐TOM) clusters. Gene expression similarity was assessed by a pairwise weighted correlation measure and clustered into modules according to a topological overlap measure. Each colour represents a co‐expression module, and each branch represents a gene. (e) Heatmap of module signature gene associations with the CAVD disease status. The correlation coefficient and *p*‐value are labelled in each cell, and the colour type and shade of each cell represent the strength of the correlation. CAVD, calcific aortic valve disease.

We next selected the top 25% of DEGs from the merged CAVD datasets for WGCNA analysis. As shown in Figure 5a, a sample clustering tree, including all samples, was well obtained. Then, the optimal soft threshold of 20 (based on scale‐free *R* ^2^ = 0.85; Figures 5b,c) was used to obtain the modules for further analysis, and a total of four modules were identified (Figure 5d). A correlation analysis was then performed to explore the importance of modular genes in CAVD development. The strongest positive correlation was observed in the turquoise module (*r* = 0.83, *P* = 2e‐08), while the grey module had a significant negative correlation (*r* = −0.8, *P* = 1e‐07) (Figure 5e). Because the biological functions of DEGs in merged CAVD GEO datasets were strongly associated with inflammatory responses in CAVD, we then selected the turquoise module as the key module for further analyses.

### 3.4 Identification of hub genes through machine learning

**FIGURE 6:**
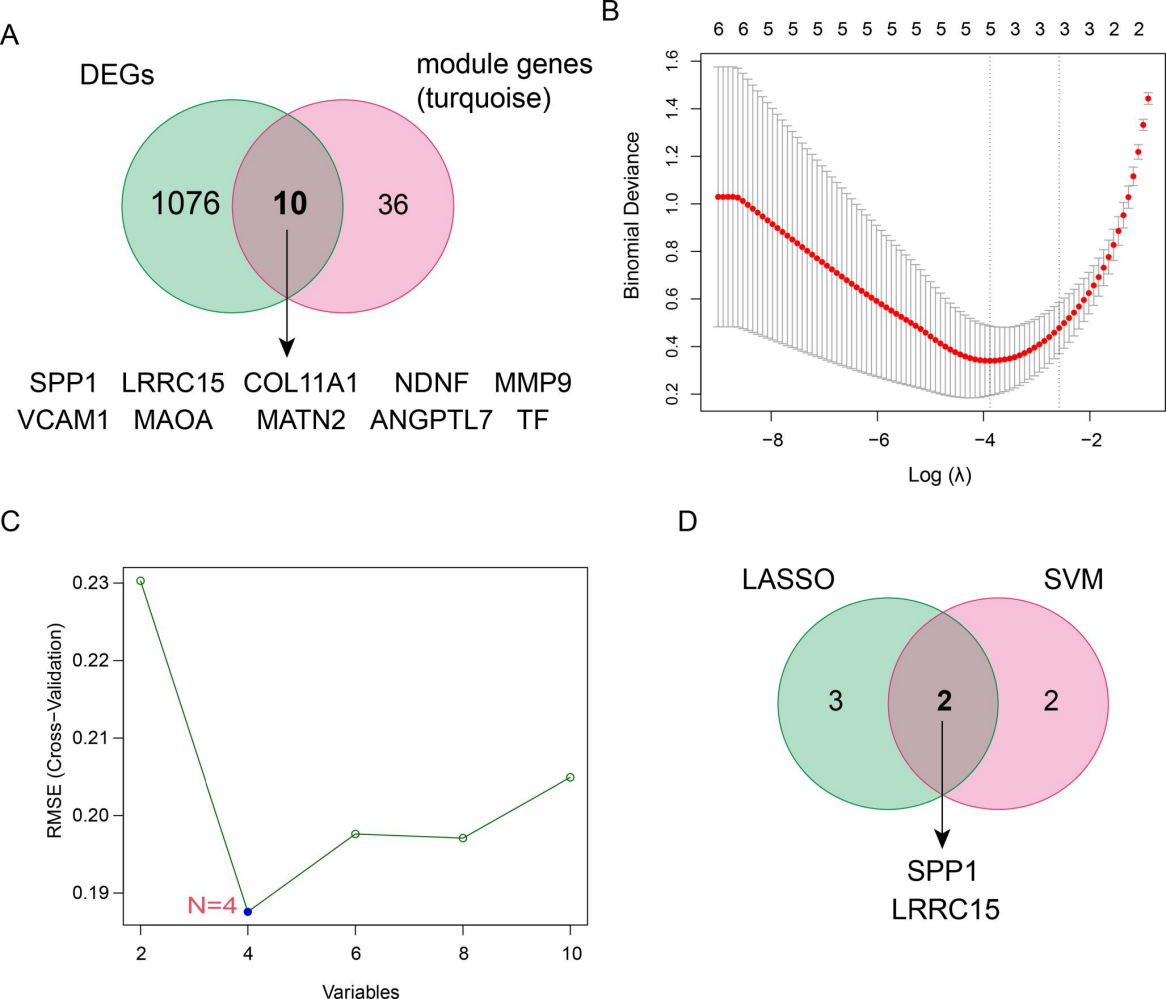
Screening of candidate genes by machine learning method. (a) Vene plot of calcific aortic valve disease key module genes and osteoarthritis differentially expressed genes. (b) Screening of candidate biomarkers by the LASSO model. The ordinate is the value of the coefficient, the lower abscissa is log(*λ*), and the upper abscissa is the number of non‐zero coefficients in the model. (c) Screening of candidate biomarkers by the SVM‐RFE algorithm. (d) Vene plot of candidate genes screened by LASSO and SVM‐RFE. LASSO, least absolute shrinkage sum selection operator; SVM‐RFE, support vector machine‐recursive feature elimination.

The turquoise module contained 36 genes (Figure 5e). By combining those key module genes and DEGs of OA (Figure 3c), we identified 10 overlapped common genes (Figure 6a). Moreover, we used two machine learning methods, including LASSO and SVM‐RFE algorithms, separately to further discover the key hub genes among those 10 overlapped common genes. The LASSO algorithm identified five potential candidates, including LRRC15, SPP1, angiopoietin like 7 (ANGPTL7), matrilin 2 (MATN2), and transferrin (TF) (Figure 6b), whereas the SVM‐RFE algorithm identified LRRC15, SPP1, monoamine oxidase A (MAOA), and collagen type XI alpha 1 chain (COL11A1) as the possible hub genes (Figure 6c). Thus, we selected LRRC15 and SPP1 as the key hub genes that may play critical roles in inflammation involved in the pathogenesis of CAVD and OA (Figure 6d).

**FIGURE 7:**
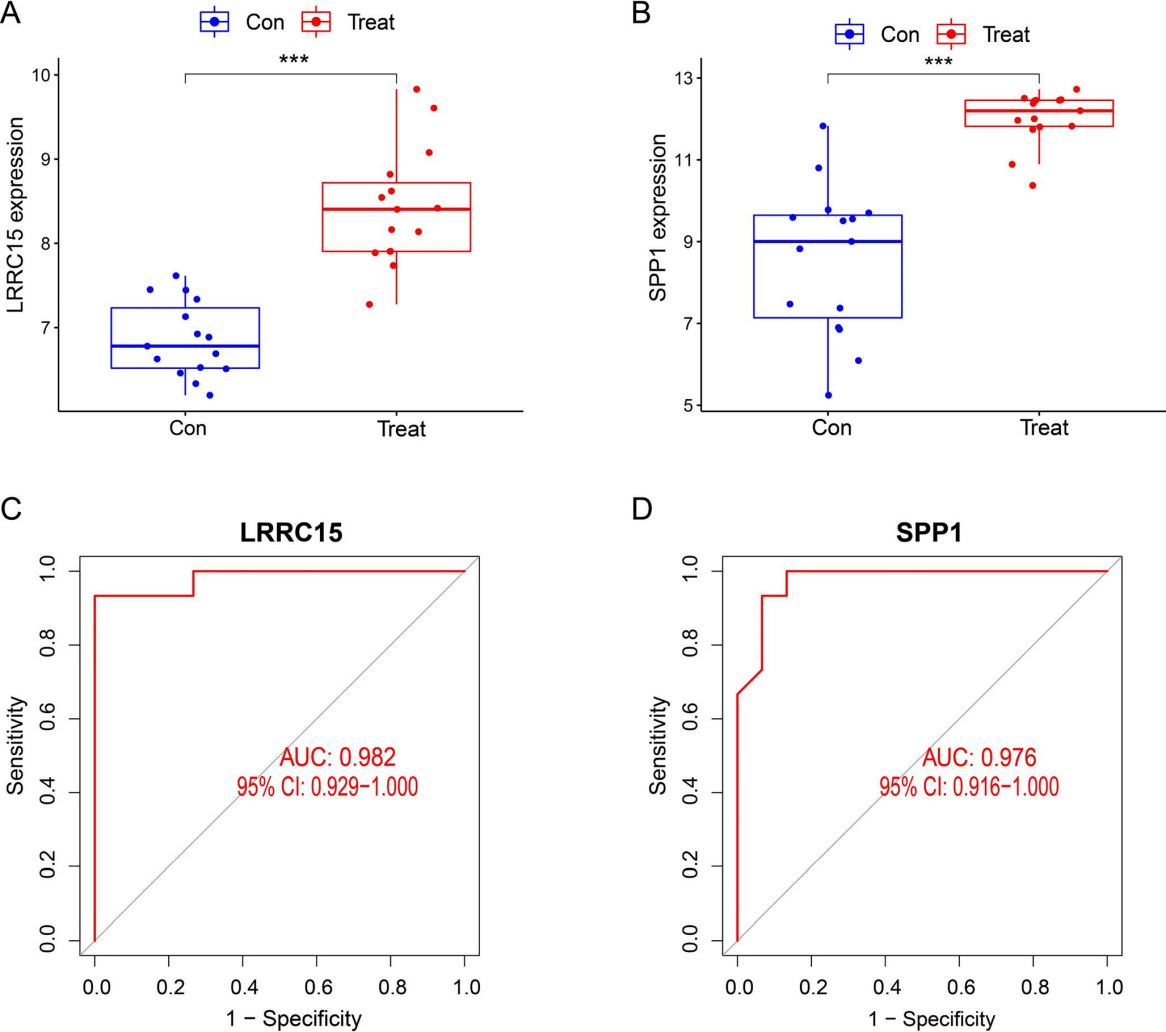
Validation of candidate biomarkers in the combined GSE12644 and GSE51472 datasets. (a) Expression of LRRC15 in CAVD and normal groups. (b) Expression of secreted phosphoprotein 1 in CAVD and normal groups. (c) Diagnostic performance of LRRC15 expression in CAVD. ***p < 0.001 (d) Diagnostic performance of SPP1 expression in CAVD. AUC, area under the ROC curve; CAVD, calcific aortic valve disease; LRRC15, leucine rich repeat containing 15; ROC, receiver operating characteristic.

**FIGURE 8:**
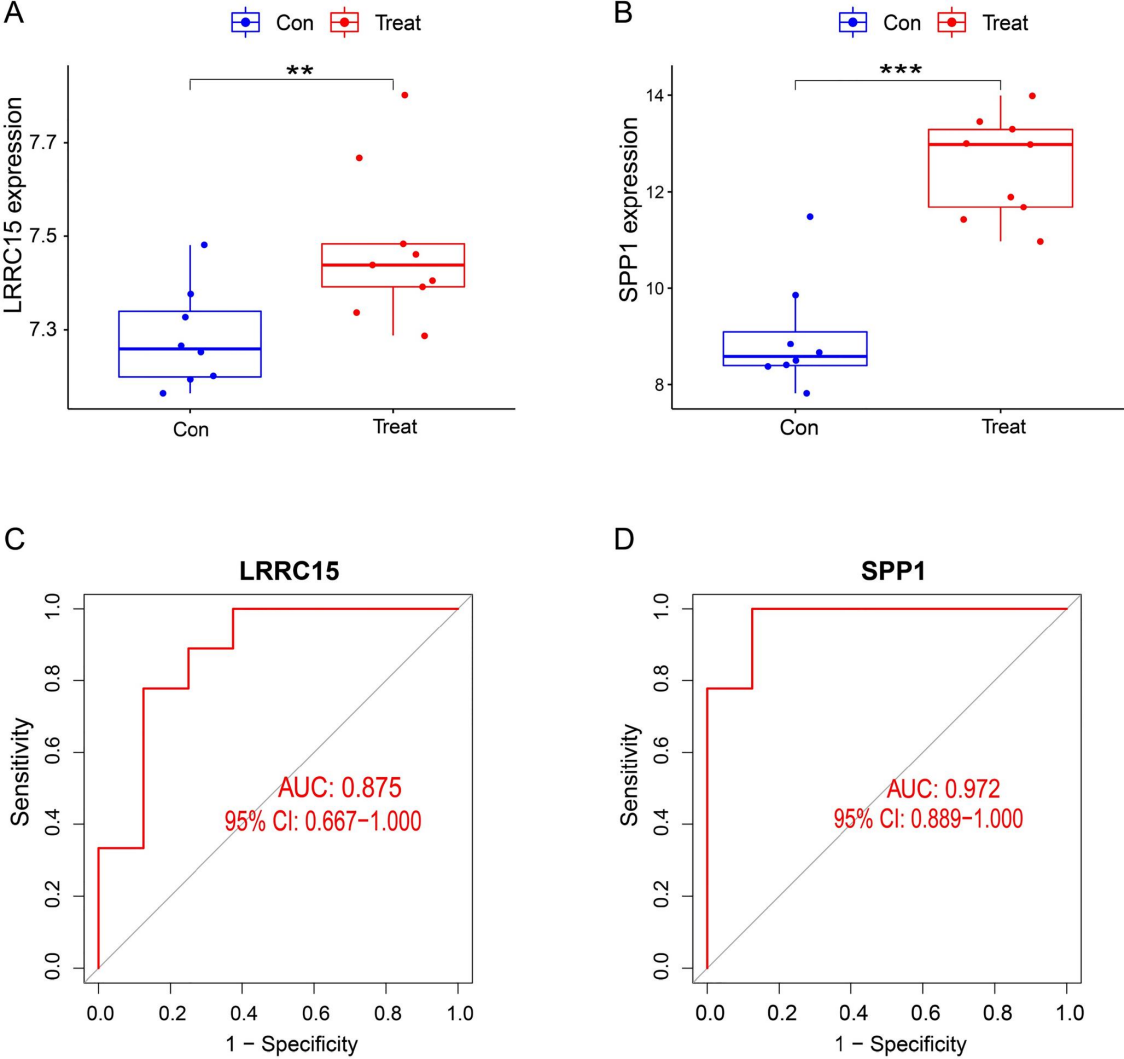
Validation of candidate hub genes in the validation dataset GSE83453. (a) Expression of LRRC15 in CAVD and normal groups. (b) Expression of SPP1 in CAVD and normal groups. (c) Diagnostic performance of LRRC15 expression in CAVD. **p < 0.01, ***p < 0.001 (d) Diagnostic performance of SPP1 expression in CAVD. AUC, area under the ROC curve; CAVD, Calcific aortic valve disease; LRRC15, leucine rich repeat containing 15; ROC, receiver operating characteristic; SPP1, secreted phosphoprotein 1.

**FIGURE 9:**
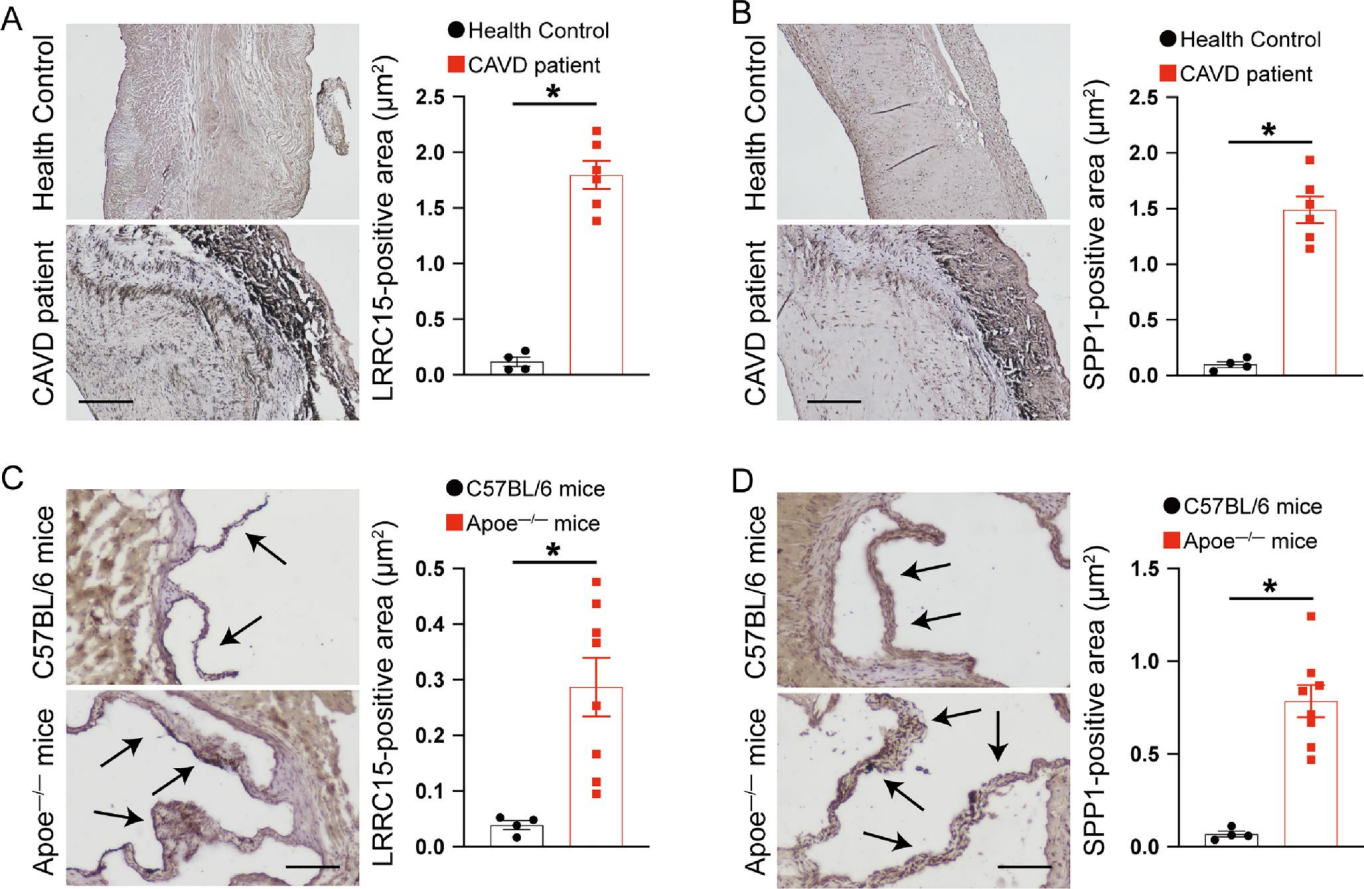
Validation of candidate hub genes in the human and mice calcific aortic valve tissues. (a) Expression of LRRC15 in aortic valves of health subjects and CAVD patients. (*n* = four to six per group). Scale: 400 *μ*m. (b) Expression of SPP1 in aortic valves of health subjects and CAVD patients. (*n* = four to six per group). Scale: 400 *μ*m. (c) Expression of LRRC15 in aortic valves of age‐matched C57BL/6 and *Apoe* ^*−/−*^ mice. Black arrow indicates SPP1‐positive area in aortic valves (*n* = four to eight mice per group). Scale: 100 μm. (d) Expression of SPP1 in aortic valves of age‐matched C57BL/6 and *Apoe* ^*−/−*^ mice. Black arrow indicates the SPP1‐positive area in aortic valves (*n* = four to eight mice per group). Scale: 100 *μ*m. Data shown are mean ± SEM. *p < 0.05. CAVD, Calcific aortic valve disease; LRRC15, leucine rich repeat containing 15; SPP1, secreted phosphoprotein 1.

As shown in Figure 7a,b, the expressions of LRRC15 and SPP1 were significantly increased in the CAVD group compared to the normal group. Moreover, those two genes exhibited significant discriminative power in distinguishing CAVD from controls, with an AUC of 0.982 (95% CI: 0.929–1.000) for LRRC15 and an AUC of 0.976 (95% CI: 0.916–1.000) for SPP1 (Figure 7c,d). These observations (Figure 7) were further confirmed by using the GSE83453 dataset to evaluate the diagnostic effect (Figure 8). Furthermore, immunohistochemistry staining showed that LRRC15 and SPP1 expressions in aortic valve tissues were significantly increased in both CAVD patients and high‐cholesterol diet‐induced experimental CAVD mice using apolipoprotein e‐deficient mice (*Apoe* ^*−/−*^) compared to health control subjects and normal chow‐diet‐fed wild‐type C57BL/6 mice (Figure 9).

### 3.5 Immune cell infiltration analysis

**FIGURE 10:**
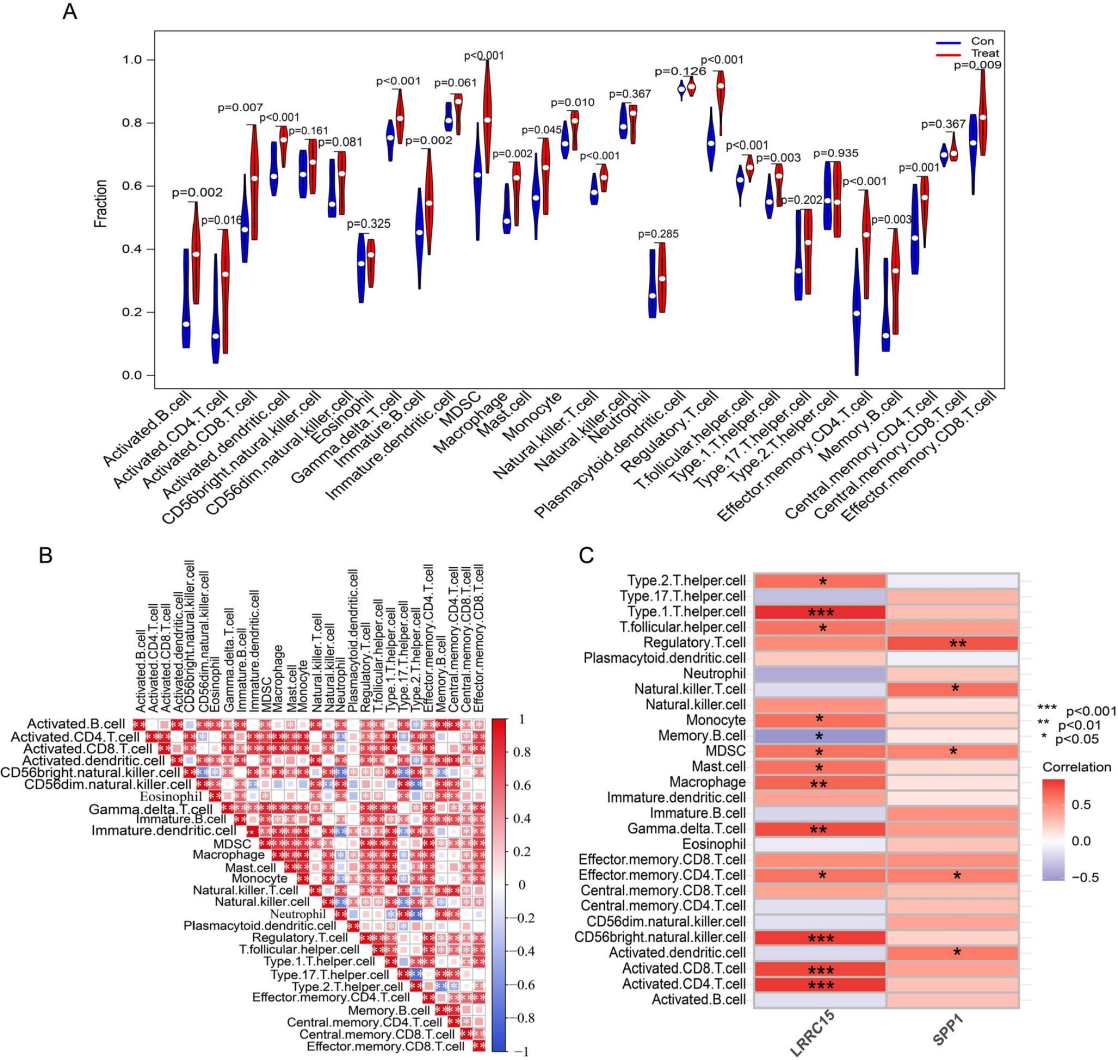
CAVD immune infiltration and its correlation with LRRC15, SPP1 expression. (a) Comparison of various types of immune cell infiltration between normal and CAVD groups. Blue represents the normal group, red represents the CAVD group, and the *p* value is marked above. (b) Correlation composition of various immune cell types. *p < 0.05, **p < 0.01, ***p < 0.001. (c) Visualisation of the correlation analysis results of LRRC15 and SPP1 expression and immune cell infiltration. The colour of each cell represents the magnitude of the correlation coefficient, blue represents the negative correlation, and red represents the positive correlation. Asterisks represent *p* values, *p < 0.05, **p < 0.01, ***p < 0.001. CAVD, Calcific aortic valve disease; LRRC15, leucine rich repeat containing 15; SPP1, secreted phosphoprotein 1.

The ssGSEA method was used to analyse the immune cells infiltration between the CAVD and control group. As shown in Figure 10a, a significantly higher fraction of B cells, CD4‐positive T cells, CD8‐positive T cells, macrophages, and NK cells was observed in the CAVD group than that in normal controls. Figure 10b exhibited the correlation between the individual immune cells. Moreover, we also investigated the association between individual immune cells and hub genes. We found that the expression of LRRC15 was significantly positively correlated with the infiltration of type 1 T helper cells, CD56 NK cells, activated CD8 T cells, and activated CD4 T cells, while it was negatively correlated with the infiltration of memory B cells (Figure 10c). SPP1 had strong positive correlations with regulatory T cells, NK T cells, MDSC, effector memory CD4 T cells and activated dendritic cell (Figure 10c).

### 3.6 The ceRNA network analysis of hub genes

**FIGURE 11:**
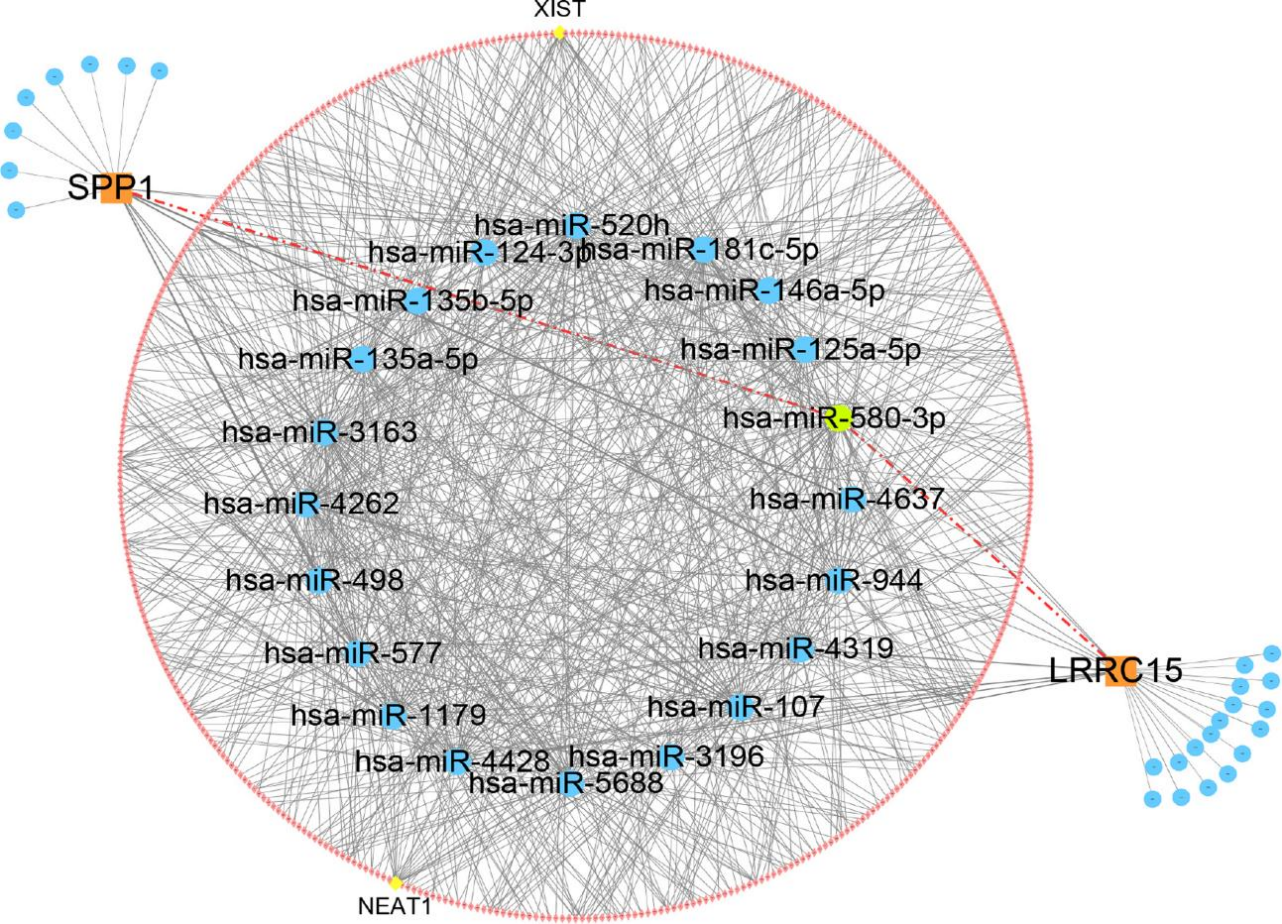
A ceRNA networks based on hub genes. The network included 480 nodes (2 hub genes, 45 miRNAs and 433 lncRNAs) and 887 edges.

We also generated a ceRNA network on those two hub genes, LRRC15 and SPP1. As shown in Figure 11, a total of 480 points (including 2 hub genes, 45 miRNAs and 433 lncRNAs) and 887 edges were identified in the ceRNA network. Specifically, 399 lncRNAs regulate LRRC15 through 10 miRNAs, and 442 lncRNAs competitively bind various miRNAs (such as hsa‐miR‐181c‐5p, hsa‐miR‐520h, hsa‐miR‐3163 and hsa‐miR‐4262) to regulated SPP1 expression. Moreover, we identified that hsa‐miR‐580‐3p regulated both LRRC15 and SPP1. Furthermore, we found that LncRNA NEAT1 and lncRNA XIST regulated 19 and 18 miRNAs, respectively, suggesting that they were at the core of the network.

## 4 DISCUSSION

Inflammation contributes importantly to CAVD and OA that affect adults aged 60 years and older worldwide [10, 29, 30]. Due to the lack of effective drugs to limit the progression of CAVD and OA, discovering the underlying mechanisms and developing potential novel therapeutics is critical for patients with CAVD and OA. In this study, by using WGCNA and machine learning methods, we integrated multiple CAVD and OA datasets from GEO and identified that LRRC15 and SPP1 exhibited a high possibility of serving as novel targets. Moreover, bioinformatics analytic methods revealed that LRRC15 and SPP1 play important roles in modulating multiple inflammatory processes that affected CAVD development, including leucocyte recruitment, migration, infiltration and activation. Furthermore, ceRNA network analysis showed that lncRNA NEAT1 and XIST were key lncRNAs in the network, and hsa‐miR‐580‐3p regulated both LRRC15 and SPP1 expressions. Together, our findings suggested that LRRC15 and SPP1 may be crucial in immunological mechanisms during CAVD and OA initiation and progression, as we potential targets for drug development.

A total of 544 DEGs (311 upregulated and 233 downregulated genes) were identified in the merged CAVD cohort (Figure 3a,b). Consistent with previous studies [31, 32, 33], GO and KEGG enrichment analysis revealed that multiple inflammation associated processes were activated in CAVD group, such as leukocyte chemotaxis, leukocyte migration, cell chemotaxis, cytokine activity, cytokine‐cytokine receptor and chemokine signalling pathways were significantly enriched (Figure 4). Indeed, inflammatory cytokines and their actions on cytokine receptors play critical roles in regulating immune cells infiltration and activation, which eventually result in aortic valve fibrosis and calcification [16, 34, 35, 36]. For example, Winchester et al. found that patients with aortic stenosis (AS) developed a sustained systemic adaptive immune response, including the activation of circulating CD8 T cells [34]. Moreover, macrophages, CD4‐ and CD8‐positve T cells, B cells and NK cells are positively associated with pressure gradients and aortic valve calcification [35, 37, 38, 39, 40]. In line with those observations, ssGSEA analysis showed a significant increase of such immune cells in the CAVD group (Figure 10a).

One essential question that needs to be figured out is to identify the potential key genes controlling inflammatory processes. We identified two potential hub genes, LRCC15 and SPP1, by using WGCNA analysis and machine learning. The type I transmembrane protein LRRC15 belongs to the LRR family, and the major function of the LRR family is to facilitate protein‐protein and protein‐matrix interactions [41, 42]. Accumulating evidence demonstrated that LRRC15 was abundantly expressed in mineralised tissues and was upregulated in response to multiple proinflammatory cytokines, such as TNF‐*α*, IL‐1*β* or IFN‐*γ* [43, 44]. High expression of LRRC15 was associated with the increased infiltration of macrophages, and macrophages are the key immune cells that promoted aortic valve calcification [45, 46]. Moreover, Wang et al. showed that LRRC15 promoted mesenchymal stem cells osteogenic differentiation [47], a characteristic pathological alteration that exhibited in sclerotic regions of human advanced OA [48]. In the current study, KEGG enrichment analyses indicated that IL‐17 and NF‐kappa B signalling pathways were significantly affected. Indeed, IL‐17 and NF‐kappa B signalling pathways play critical roles in controlling TNF‐*α* and IL‐*β* production and secretion in macrophages [49]. Moreover, the expression of LRRC15 was positively correlated with multiple immune cells (Figure 10c). These data suggest that LRRC15 regulates macrophage activation through IL‐17 and NF‐kappa B signaling pathways and subsequently contributes to CAVD and OA development, but further validation experiments are required.

In addition to LRRC15, SPP1, also known as osteopontin, was highly expressed in the CAVD group (Figure 7 to Figure 9) and this was consistent with previous studies [27, 50, 51, 52]. SPP1 was initially implicated in bone mineralisation and was subsequently shown to be a pro‐inflammatory cytokine [52]. For instance, SPP1 induced matrix metalloproteinase 13 production and plays an important role in type II collagen degradation [53, 54]. In support of our findings, previous studies identified that SPP1 is strongly associated with the severity of knee OA and may serve as a key regulator gene in CAVD [55, 56, 57, 58]. In addition, we further identified that SPP1 was positively correlated with various immune cells (Figure 10c), suggesting that SPP1 holds a high possibility of serving as a target for the treatment of CAVD and OA.

Another critical question is the regulator mechanisms involved in those two hub genes expression. Emerging studies have shown that non‐coding RNAs play an important role in the development and progression of CAVD and OA through the modulating expression of large gene networks [59, 60]. Therefore, we intended to establish a ceRNA network map potentially in regulating LRRC15 and SPP1 expressions. Previous studies identified LINC00702‐miR‐181b‐5p axis, miR‐127‐5p and miR‐186 affects SPP1 expression in CAVD or OA [54, 57, 61], to the opposite, we identified that lncRNA NEAT1 and lncRNA XIST were key lncRNAs in the network and hsa‐miR‐580‐3p regulated both LRRC15 and SPP1 expression (Figure 11). These data suggested that lncRNA NEAT1 [62], XIST and hsa‐miR‐580‐3p may serve as potential targets for CAVD and OA treatment.

### 4.1 Limitations

There are several limitations to our study. First, although the datasets were merged, the sample size of the dataset used for analysis was relatively small. Second, further studies need to verify the key hub genes in a larger cohort and to investigate the biological functions of the candidate genes, although we examined LCRR15 and SPP1 expression in an independent CAVD dataset and in human and mice calcific aortic valves.

## 5 CONCLUSIONS

In summary, we identified that LRRC15 and SPP1 could serve as inflammation‐associated candidate hub genes or potential therapeutic targets in CAVD and OA using bioinformatics and machine learning. Given that still lacking effective medications to slow CAVD and OA progression, those findings may provide new insights for the development of novel medications for CAVD and OA.

## AUTHOR CONTRIBUTIONS

**Shuji Gong**: Conceptualization; writing – original draft. **Kun Xiang**: Visualization. **Le Chen**: Supervision. **Huanwei Zhuang**: Methodology. **Yaning Song**: Software. **Jinlan Chen**.

## CONFLICT OF INTEREST STATEMENT

The authors declare no conflicts of interest relevant to this work.

## Data Availability

All data relevant to the study are included in the article. Additional data are available from the corresponding authors upon reasonable request.

## References

[1] Iung, B. , Vahanian, A. : Epidemiology of valvular heart disease in the adult. Nat. Rev. Cardiol. 8(3), 162–172 (2011). 10.1038/nrcardio.2010.202 21263455

[2] Broeders, W. , et al.: Innate immune cells in the pathophysiology of calcific aortic valve disease: lessons to be learned from atherosclerotic cardiovascular disease? Basic Res. Cardiol. 117(1), 28 (2022). 10.1007/s00395-022-00935-6 35581364 PMC9114076

[3] Yadgir, S. , et al.: Global, regional, and national burden of calcific aortic valve and degenerative mitral valve diseases, 1990‐2017. Circulation 141(21), 1670–1680 (2020). 10.1161/circulationaha.119.043391 32223336

[4] Yutzey, K.E. , et al.: Calcific aortic valve disease: a consensus summary from the alliance of investigators on calcific aortic valve disease. Arterioscler. Thromb. Vasc. Biol. 34(11), 2387–2393 (2014). 10.1161/atvbaha.114.302523 25189570 PMC4199903

[5] Glyn‐Jones, S. , et al.: Osteoarthritis. Lancet 386(9991), 376–387 (2015). 10.1016/s0140-6736(14)60802-3 25748615

[6] Abramoff, B. , Caldera, F.E. : Osteoarthritis: pathology, diagnosis, and treatment options. Med. Clin. 104(2), 293–311 (2020). 10.1016/j.mcna.2019.10.007 32035570

[7] Cavaco, S. , et al.: Gla‐rich protein is involved in the cross‐talk between calcification and inflammation in osteoarthritis. Cell. Mol. Life Sci. 73(5), 1051–1065 (2016). 10.1007/s00018-015-2033-9 26337479 PMC11108449

[8] Myasoedova, V.A. , et al.: Novel pharmacological targets for calcific aortic valve disease: prevention and treatments. Pharmacol. Res. 136, 74–82 (2018). 10.1016/j.phrs.2018.08.020 30149054

[9] Martel‐Pelletier, J. , et al.: Osteoarthritis. Nat. Rev. Dis. Prim. 2(1), 16072 (2016). 10.1038/nrdp.2016.72 27734845

[10] Towler, D.A. : Molecular and cellular aspects of calcific aortic valve disease. Circ. Res. 113(2), 198–208 (2013). 10.1161/circresaha.113.300155 23833294 PMC4057916

[11] Mathieu, P. , Boulanger, M.C. : Basic mechanisms of calcific aortic valve disease. Can. J. Cardiol. 30(9), 982–993 (2014). 10.1016/j.cjca.2014.03.029 25085215

[12] Goody, P.R. , et al.: Aortic valve stenosis: from basic mechanisms to novel therapeutic targets. Arterioscler. Thromb. Vasc. Biol. 40(4), 885–900 (2020). 10.1161/ATVBAHA.119.313067 32160774

[13] Otto, C.M. , et al.: Characterization of the early lesion of 'degenerative' valvular aortic stenosis. Histological and immunohistochemical studies. Circulation 90(2), 844–853 (1994). 10.1161/01.cir.90.2.844 7519131

[14] Peeters, F. , et al.: Calcific aortic valve stenosis: hard disease in the heart: a biomolecular approach towards diagnosis and treatment. Eur. Heart J. 39(28), 2618–2624 (2018). 10.1093/eurheartj/ehx653 29136138 PMC6055545

[15] Mohler, E.R., 3rd , et al.: Bone formation and inflammation in cardiac valves. Circulation 103(11), 1522–1528 (2001). 10.1161/01.cir.103.11.1522 11257079

[16] Steiner, I. , et al.: Calcific aortic valve stenosis: immunohistochemical analysis of inflammatory infiltrate. Pathol. Res. Pract. 208(4), 231–234 (2012). 10.1016/j.prp.2012.02.009 22436689

[17] Woodell‐May, J.E. , Sommerfeld, S.D. : Role of inflammation and the immune system in the progression of osteoarthritis. J. Orthop. Res. 38(2), 253–257 (2020). 10.1002/jor.24457 31469192

[18] Thomson, A. , Hilkens, C.M.U. : Synovial macrophages in osteoarthritis: the key to understanding pathogenesis? Front. Immunol. 12, 678757 (2021). 10.3389/fimmu.2021.678757 34211470 PMC8239355

[19] Wang, C. , et al.: Cardamonin inhibits osteogenic differentiation of human valve interstitial cells and ameliorates aortic valve calcification via interfering in the NF‐κB/NLRP3 inflammasome pathway. Food Funct. 12(23), 11808–11818 (2021). 10.1039/d1fo00813g 34766179

[20] Kloppenburg, M. : Inflammation is a relevant treatment target in osteoarthritis. Lancet (London, England) 402(10414), 1725–1726 (2023). 10.1016/S0140-6736(23)01726-9 37839421

[21] Wang, J. , Xue, Y. , Zhou, L. : Comparison of immune cells and diagnostic markers between spondyloarthritis and rheumatoid arthritis by bioinformatics analysis. J. Transl. Med. 20(1), 196 (2022). 10.1186/s12967-022-03390-y 35509008 PMC9066892

[22] Ye, Q. , et al.: Identification of the common differentially expressed genes and pathogenesis between neuropathic pain and aging. Front. Neurosci. 16, 994575 (2022). 10.3389/fnins.2022.994575 36340779 PMC9626798

[23] Sun, J.Y. , et al.: Identification of key genes in calcific aortic valve disease via weighted gene co‐expression network analysis. BMC Med. Genom. 14(1), 135 (2021). 10.1186/s12920-021-00989-w PMC8138987 34020624

[24] Xiao, S. , et al.: Uncovering potential novel biomarkers and immune infiltration characteristics in persistent atrial fibrillation using integrated bioinformatics analysis. Math. Biosci. Eng. 18(4), 4696–4712 (2021). 10.3934/mbe.2021238 34198460

[25] Subramanian, A. , et al.: Gene set enrichment analysis: a knowledge‐based approach for interpreting genome‐wide expression profiles. Proc. Natl. Acad. Sci. U.S.A. 102(43), 15545–15550 (2005). 10.1073/pnas.0506580102 16199517 PMC1239896

[26] Hu, K. : Become competent within one day in generating boxplots and violin plots for a novice without prior R experience. Methods Protoc 3(4), 64 (2020). 10.3390/mps3040064 32977580 PMC7712237

[27] Zhou, Y. , et al.: Liraglutide attenuates aortic valve calcification in a high‐cholesterol‐diet‐induced experimental calcific aortic valve disease model in apolipoprotein E‐deficient mice. J Cardiovasc Dev Dis 10(9), 386 (2023). 10.3390/jcdd10090386 37754815 PMC10531705

[28] Yang, D. , et al.: Dihydromyricetin increases endothelial nitric oxide production and inhibits atherosclerosis through microRNA‐21 in apolipoprotein E‐deficient mice. J. Cell Mol. Med. 24(10), 5911–5925 (2020). 10.1111/jcmm.15278 32301289 PMC7214150

[29] Collaborators, G.B.D.O. , et al.: Global, regional, and national burden of osteoarthritis, 1990‐2020 and projections to 2050: a systematic analysis for the Global Burden of Disease Study 2021. Lancet Rheumatol 5(9), e508–e522 (2023). 10.1016/S2665-9913(23)00163-7 37675071 PMC10477960

[30] Liberale, L. , et al.: Inflammation, aging, and cardiovascular disease: JACC review topic of the week. J. Am. Coll. Cardiol. 79(8), 837–847 (2022). 10.1016/j.jacc.2021.12.017 35210039 PMC8881676

[31] Liu, F.Y. , et al.: Role of interleukin 17A in aortic valve inflammation in apolipoprotein E‐deficient mice. Curr Med Sci 40(4), 729–738 (2020). 10.1007/s11596-020-2230-0 32862384

[32] Kapelouzou, A. , et al.: Differential expression patterns of Toll like Receptors and Interleukin‐37 between calcific aortic and mitral valve cusps in humans. Cytokine 116, 150–160 (2019). 10.1016/j.cyto.2019.01.009 30716659

[33] Syväranta, S. , et al.: Potential pathological roles for oxidized low‐density lipoprotein and scavenger receptors SR‐AI, CD36, and LOX‐1 in aortic valve stenosis. Atherosclerosis 235(2), 398–407 (2014). 10.1016/j.atherosclerosis.2014.05.933 24929820

[34] Winchester, R. , et al.: Circulating activated and effector memory T cells are associated with calcification and clonal expansions in bicuspid and tricuspid valves of calcific aortic stenosis. J. Immunol. 187(2), 1006–1014 (2011). 10.4049/jimmunol.1003521 21677140 PMC3131440

[35] Natorska, J. , et al.: Presence of B cells within aortic valves in patients with aortic stenosis: relation to severity of the disease. J. Cardiol. 67(1), 80–85 (2016). 10.1016/j.jjcc.2015.05.002 26068299

[36] Milutinovic, A. , et al.: Mast cells might have a protective role against the development of calcification and hyalinisation in severe aortic valve stenosis. Folia Biol. 62(4), 160–166 (2016) 10.14712/fb2016062040160 27643581

[37] Zhou, P. , et al.: Interleukin 37 suppresses M1 macrophage polarization through inhibition of the Notch1 and nuclear factor kappa B pathways. Front. Cell Dev. Biol. 8, 56 (2020). 10.3389/fcell.2020.00056 32117982 PMC7033589

[38] Nagy, E. , et al.: Interferon‐γ released by activated CD8(+) T lymphocytes impairs the calcium resorption potential of osteoclasts in calcified human aortic valves. Am. J. Pathol. 187(6), 1413–1425 (2017). 10.1016/j.ajpath.2017.02.012 28431214 PMC5455058

[39] Mazur, P. , et al.: Lymphocyte and monocyte subpopulations in severe aortic stenosis at the time of surgical intervention. Cardiovasc. Pathol. 35, 1–7 (2018). 10.1016/j.carpath.2018.03.004 29727769

[40] Raddatz, M.A. , et al.: Macrophages promote aortic valve cell calcification and alter STAT3 splicing. Arterioscler. Thromb. Vasc. Biol. 40(6), e153–e165 (2020). 10.1161/atvbaha.120.314360 32295422 PMC7285853

[41] Kobe, B. , Deisenhofer, J. : The leucine‐rich repeat: a versatile binding motif. Trends Biochem. Sci. 19(10), 415–421 (1994). 10.1016/0968-0004(94)90090-6 7817399

[42] Kobe, B. , Kajava, A.V. : The leucine‐rich repeat as a protein recognition motif. Curr. Opin. Struct. Biol. 11(6), 725–732 (2001). 10.1016/s0959-440x(01)00266-4 11751054

[43] Cooper, P.R. , et al.: Mediators of inflammation and regeneration. Adv. Dent. Res. 23(3), 290–295 (2011). 10.1177/0022034511405389 21677081

[44] Satoh, K. , Hata, M. , Yokota, H. : A novel member of the leucine‐rich repeat superfamily induced in rat astrocytes by beta‐amyloid. Biochem. Biophys. Res. Commun. 290(2), 756–762 (2002). 10.1006/bbrc.2001.6272 11785964

[45] Li, G. , et al.: The shift of macrophages toward M1 phenotype promotes aortic valvular calcification. J. Thorac. Cardiovasc. Surg. 153(6), 1318.e1–1327.e1 (2017). 10.1016/j.jtcvs.2017.01.052 28283241

[46] Tang, H. , et al.: Integrated microenvironment‐associated genomic profiles identify LRRC15 mediating recurrent glioblastoma‐associated macrophages infiltration. J. Cell Mol. Med. 25(12), 5534–5546 (2021). 10.1111/jcmm.16563 33960636 PMC8184692

[47] Wang, Y. , et al.: LRRC15 promotes osteogenic differentiation of mesenchymal stem cells by modulating p65 cytoplasmic/nuclear translocation. Stem Cell Res. Ther. 9(1), 65 (2018). 10.1186/s13287-018-0809-1 29523191 PMC5845373

[48] Ilas, D.C. , et al.: The simultaneous analysis of mesenchymal stem cells and early osteocytes accumulation in osteoarthritic femoral head sclerotic bone. Rheumatology 58(10), 1777–1783 (2019). 10.1093/rheumatology/kez130 31165896 PMC6758575

[49] Jovanovic, D.V. , et al.: IL‐17 stimulates the production and expression of proinflammatory cytokines, IL‐beta and TNF‐alpha, by human macrophages. J. Immunol. 160(7), 3513–3521 (1998). 10.4049/jimmunol.160.7.3513 9531313

[50] Mohler, E.R., 3rd , et al.: Detection of osteopontin in calcified human aortic valves. Arterioscler. Thromb. Vasc. Biol. 17(3), 547–552 (1997). 10.1161/01.atv.17.3.547 9102175

[51] Greene, C.L. , et al.: Transcriptional profiling of normal, stenotic, and regurgitant human aortic valves. Genes 11(7), 789 (2020). 10.3390/genes11070789 32674273 PMC7397246

[52] Passmore, M. , et al.: Osteopontin alters endothelial and valvular interstitial cell behaviour in calcific aortic valve stenosis through HMGB1 regulation. Eur. J. Cardio. Thorac. Surg. 48(3), e20–e29 (2015). 10.1093/ejcts/ezv244 26273067

[53] Cheng, C. , Gao, S. , Lei, G. : Association of osteopontin with osteoarthritis. Rheumatol. Int. 34(12), 1627–1631 (2014). 10.1007/s00296-014-3036-9 24807695

[54] Liang, J. , et al.: MALAT1/miR‐127‐5p regulates osteopontin (OPN)‐Mediated proliferation of human chondrocytes through PI3K/akt pathway. J. Cell. Biochem. 119(1), 431–439 (2018). 10.1002/jcb.26200 28590075

[55] Lok, Z.S.Y. , Lyle, A.N. : Osteopontin in vascular disease. Arterioscler. Thromb. Vasc. Biol. 39(4), 613–622 (2019). 10.1161/atvbaha.118.311577 30727754 PMC6436981

[56] Qiao, E. , Huang, Z. , Wang, W. : Exploring potential genes and pathways related to calcific aortic valve disease. Gene 808, 145987 (2022). 10.1016/j.gene.2021.145987 34600049

[57] Huang, K. , et al.: Transcriptome sequencing data reveal LncRNA‐miRNA‐mRNA regulatory network in calcified aortic valve disease. Front Cardiovasc Med 9, 886995 (2022). 10.3389/fcvm.2022.886995 35722091 PMC9204424

[58] Honsawek, S. , et al.: Correlation of plasma and synovial fluid osteopontin with disease severity in knee osteoarthritis. Clin. Biochem. 42(9), 808–812 (2009). 10.1016/j.clinbiochem.2009.02.002 19217889

[59] Gupta, S.K. , et al.: Non‐coding RNAs: regulators of valvular calcification. J. Mol. Cell. Cardiol. 142, 14–23 (2020). 10.1016/j.yjmcc.2020.03.015 32247640

[60] Ni, W.J. , et al.: Noncoding RNAs in calcific aortic valve disease: a review of recent studies. J. Cardiovasc. Pharmacol. 71(5), 317–323 (2018). 10.1097/fjc.0000000000000569 29734266

[61] Lin, Z. , et al.: microRNA‐186 inhibition of PI3K‐AKT pathway via SPP1 inhibits chondrocyte apoptosis in mice with osteoarthritis. J. Cell. Physiol. 234(5), 6042–6053 (2019). 10.1002/jcp.27225 30500068

[62] Wang, Q. , et al.: NEAT1/miR‐181c regulates osteopontin (OPN)‐Mediated synoviocyte proliferation in osteoarthritis. J. Cell. Biochem. 118(11), 3775–3784 (2017). 10.1002/jcb.26025 28379604

